# Real-world effectiveness of long-acting injections for reducing recurrent hospitalizations in patients with schizophrenia

**DOI:** 10.1186/s12991-019-0254-2

**Published:** 2020-01-14

**Authors:** Hye Ok Kim, Gi Hyeon Seo, Boung Chul Lee

**Affiliations:** 1 Health Insurance Review and Assessment Service, Seoul, South Korea; 2 Department of Psychiatry, Hangang Sacred Heart Hospital, Hallym University Medical Center, 12, Beodeunaru-ro 7-gil, Yeongdeungpo-gu, 07247 Seoul, South Korea

**Keywords:** Schizophrenia, Readmission, Long-acting injection

## Abstract

**Background:**

The comparative effectiveness of antipsychotic long-acting injections (LAIs) and oral medication is not clear due to various methodological problems.

**Methods:**

To compare the effectiveness of LAIs and oral antipsychotics in preventing readmission in patients with schizophrenia, we performed a within-subject analysis of data collected from 75,274 patients hospitalized with schizophrenia over a 10-year period (2008–2017). Readmission rates were compared according to medication status (non-medication, oral medication alone, and LAI medication). Each admission episodes were compared according to medication status before admission.

**Results:**

Total 132,028 episodes of admission were analyzed. During 255,664 person-years of total observation, 101,589 outcome events occurred. Comparing LAI to only oral medication, IRR was 0.71 (0.64–0.78, *P* < 0.001). IRR of LAI to only oral medication of first index admission was 0.74 (0.65–0.86). As hospitalization was repeated, IRR of second, third, and fourth or more index admission decreased 0.65 (0.53–0.79), 0.56 (0.43–0.76), and 0.42 (0.31–0.56), respectively.

**Conclusions:**

LAI treatment reduced the readmission rate by 29% compared with oral medication in real-world settings. Moreover, LAIs reduced the readmission rate by 58% in patients with repeated admissions. The more readmissions, the greater the effect of LAIs in reducing the risk of re-hospitalization compared with oral antipsychotics.

## Introduction

### Long-acting injections in schizophrenia

Schizophrenia is a chronic debilitating psychiatric disorder. The majority of patients experience multiple relapses during the course of the disease [1]. Continuous long-term antipsychotic medication plays an important role in controlling symptoms and preventing relapse [2, 3]. Despite the critical importance of medication, the recent Clinical Antipsychotic Trial of Intervention Effectiveness (CATIE) study found that 74% of patients discontinued medication within 18 months of starting treatment [4]. Clinical practice guidelines recommend the use of antipsychotic long-acting injections (LAIs) for non-adherent patients [5]. Given the advantages of LAIs over oral antipsychotics for non-compliant patients with schizophrenia, LAIs are not used widely as expected [6]. Previous randomized controlled trials (RCTs) comparing the effectiveness of LAIs and oral antipsychotic treatments in patients with schizophrenia have yielded variable and inconclusive results [7, 8]. Because non-adherent patients cannot participate in RCTs, those most likely to benefit from LAI treatment may be excluded from clinical trials. To overcome this problem, a recent large-scale cohort study used a within-subject design to compare the effectiveness of antipsychotic treatment in patients with schizophrenia [9]. The authors found that LAIs were associated with a 20–30% lower risk of readmission during LAI than the equivalent oral formulation.

From March 2017, the payment system of psychiatry medical aid outpatients was changed from per diem to fee for service. This change allows for more proper treatment for low-income outpatients, which could lower the high numbers of psychiatry inpatient beds in Korea. We think LAI is a useful treatment to improve compliance and help patient deinstitutionalization. The aim of our claim-based study was to investigate the effectiveness of LAIs in preventing readmissions in real-world settings in South Korea. We compared the incidence rates (IR) of readmission during non-medication, oral medication alone, and LAI treatment periods and assessed changes in IRs with repeated hospitalizations.

## Methods

We obtained data from the Health Insurance Review and Assessment Service (HIRA) database, which contains every national healthcare service claims data from patients across South Korea. Although several other types of insurance cover medical treatments, such as industrial accident insurance, most patients with schizophrenia in South Korea are covered by the National Health Insurance plan. The HIRA database includes patient demographics, diagnosis according to the International Classification of Diseases Tenth Revision (ICD 10), procedure codes, and prescriptions. Identifying data were removed from the HIRA service records in accordance with the Act on the Protection of Personal Information Maintained by Public Agencies.

**Fig. 1:**
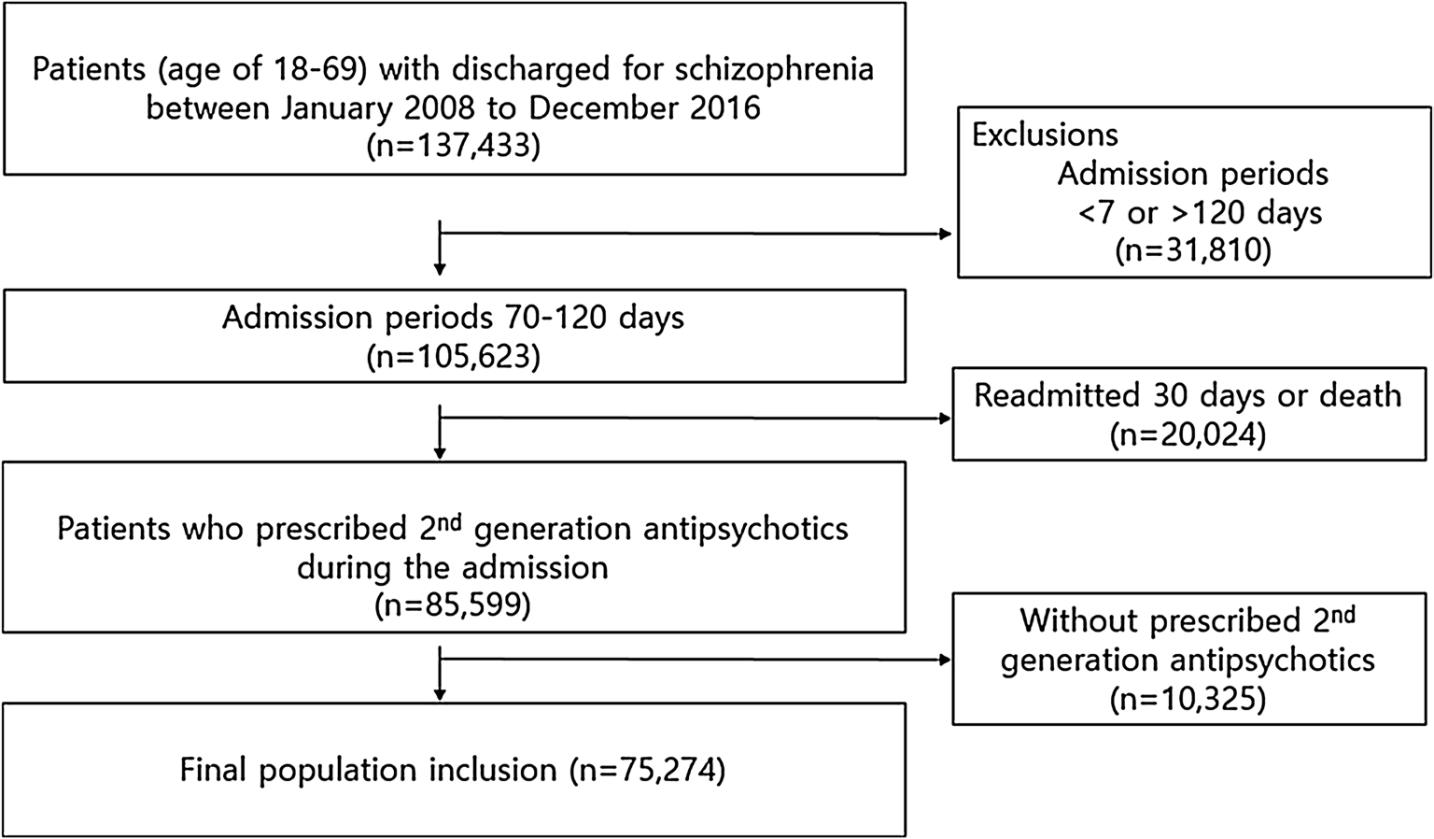
Flowchart of the study population

We used the HIRA database to identify patients (18–69 years old) diagnosed with schizophrenia (ICD10 code F20 Schizophrenia or F25 Schizoaffective disorder) who were admitted (index admission) and discharged from the hospital between January 2008 and December 2016. To ensure the enrollment of patients who received optimal in-hospital treatment, patients who did not receive second-generation antipsychotics (SGA; amisulpride, aripiprazole, olanzapine, paliperidone, quetiapine, risperidone, ziprasidone, and zotepine), or were hospitalized for less than 7 days or more than 120 days, were excluded from the study. Furthermore, patients readmitted within 30 days of discharge, which indicated inappropriate in-hospital treatment, and those admitted for non-medical reasons were excluded from the analysis (Fig. 1). In cases with multiple admissions, each admission was included and considered to be a unique episode. Prescription data and readmissions and deaths between January 2007 and December 2017 were assessed. The observation period for each episode started 30 days after discharge for every index admission. The end of the observation period was defined as the end of the study period (December 2017), readmission, or death, whichever occurred first. Re-hospitalization during the observation period was defined as an outcome event.

Each patient served as their own control to control for fixed confounding factors. The incidence rate (IR) was calculated as event count per 10 person-years. The IRs of outcome events for each period (LAIs, oral medication, and non-medication) were expressed as the incidence rate ratio (IRR). The operational definition of an oral medication period was the time from each first prescription date to last prescription date adding the last prescription duration × 1.25 (maximum refill date + 14 days). The follow-up time was reset to zero after each event to enable within individual comparisons for each episode. Each episode was analyzed separately according to the number of index admissions (first episode without previous SGA, first episode with previous SGA, two, three, and four or more episodes). We used readmission IRRs during the various periods to assess the effectiveness of LAIs (aripiprazole, paliperidone, and risperidone) compared with oral antipsychotics (amisulpride, aripiprazole, olanzapine, paliperidone, quetiapine, risperidone, ziprasidone, zotepine) in reducing multiple readmissions. Patients having oral medication and LAIs at the same time, were included to LAIs group.

Descriptive statistical analyses were performed using R software (ver. 3.4.0; R Development Core Team, Vienna, Austria). Continuous and categorical variables are expressed as mean ± standard deviation (SD), numbers (%), or medians (interquartile range; IQR). Continuous variables were compared using *t* tests, and categorical variables were compared using the Chi-square test. The outcome event rate was defined as episode counts per 10 person-years. The IRR and corresponding 95% confidence intervals (CIs) were calculated using the Poisson distribution. Two-tailed *P* < 0.05 were considered to indicate statistical significance.

## Results

### Study population characteristics

The study population characteristics are shown in Table 1. In total, we assessed 132,028 admission episodes in 75,274 patients. Their ages ranged from 18 to 69 years, with a mean age at inclusion was 40.3 ± 12.0 years. The diagnoses at discharge were F20 Schizophrenia (92.3%) and F25 Schizoaffective disorder (7.7%). The hospitals of discharge included tertiary general hospitals (13.8%), general hospitals (14.5%), and clinics or other facilities (71.8%). The median duration of hospitalization was 40 days (IQR 24–67 days). The median observation period was 369 days (IQR 103–1004 days).

**Table 1:** Study population characteristics

	Patients	Episodes
Total	75,274	132,028
Mean age (years)	40.3 ± 12.0	
Sex		
Male	35,103 (46.6%)	
Female	40,171 (53.4%)	
Diagnosis		
F20 Schizophrenia		121,884 (92.3%)
F25 Schizoaffective disorder		10,144 (7.7%)
Hospital of discharge		
Tertiary		18,182 (13.8%)
General		19,114 (14.5%)
Clinic or other		94,732 (71.8%)
Duration of admission (days)		40 (24–67)
Observation period (days)		369 (103–1004)
Sum of periods (person-years)		
All		255,664
Non-medication		80,104
Oral alone		169,948
LAI		5612
Number of episodes		
1	47,535 (63.1%)	
2	14,962 (19.9%)	
3	6200 (8.2%)	
4 or more	6577 (8.7%)	
Outcome event		101,589 (76.9%)
Death		1795
Readmission		99,794
F20 Schizophrenia		66,560 (66.7%)
F25 Schizoaffective disorder		5448 (5.5%)
F20–29 except F20 and F25		1393 (1.4%)
F00–F99 except F20-F29		5834 (5.8%)
Other codes		20,559 (20.6%)

*LAI* long-acting injection

The summed periods of non-medication, oral medication alone, and LAIs were 80,104, 169,948, and 5612 person-years, respectively. Of the 75,274 patients, 63.1% had one episode, 19.9% had two episodes, 8.2% had three episodes, and 8.7% had four or more episodes of hospitalization.

Readmission and death accounted for 75.6 and 1.6%, each of 132,028 episodes. The primary diagnosis of outcome episode was F20 Schizophrenia in 66.7% of episodes, F25 Schizoaffective disorder in 1.4% of episodes, and F00–F99 (except F20–F29) in 5.8% episodes. The primary diagnoses were other than F codes in 20.6% of episodes.

### Outcomes

The risk of a readmission for antipsychotics is shown in Table 2. In total, 101,589 outcome events (readmission or death) occurred during 255,664 person-years of observation. The IRs during the non-medication, oral medication alone, and LAI medication periods were 4.58 (4.47–4.70), 3.73 (3.67–3.79), and 2.63 (2.39–2.91), respectively. The IRRs of non-medication versus oral medication alone and non-medication versus LAI were 0.81 (0.80–0.83, *P* < 0.001) and 0.57 (0.52–0.63, *P* < 0.001), respectively. The IRR for LAI versus oral medication alone was 0.71 (0.64–0.78, *P* < 0.001).

**Table 2:** Incidence rate (IR) and incidence rate ratio (IRR)

	Non-medication	Oral medication alone		LAI medication	
Overall					
Observational period (person-years)	80,104	169,948		5612	
Outcome event (count)	36,721	63,390		1478	
1 episode					
Observational period	61,884	114,631		2895	
Outcome event	22,593	32,045		601	
2 episode					
Observational period	11,992	33,385		1476	
Outcome event	7187	13,999		401	
3 episode					
Observational period	3476	12,042		635	
Outcome event	3124	7098		213	
4 or more episode					
Observational period	2752	9889		606	
Outcome event	3817	10,248		263	
IR					
Overall	4.58 (4.47–4.70)	3.73 (3.67–3.79)		2.63 (2.39–2.91)	
1 episode without SGA before	2.68 (2.55–2.81)	2.21 (2.10–2.32)		1.57 (1.01–2.47)	
1 episode with SGA before	4.28 (4.13–4.43)	2.94 (2.87–3.01)		2.15 (1.85–2.50)	
2 episode	5.99 (5.64–6.38)	4.19 (4.04–4.36)		2.71 (2.24–3.29)	
3 episode	8.98 (8.03–10.06)	5.90 (5.55–6.27)		3.33 (2.51–4.47)	
4 or more episode	13.88 (12.28–15.74)	10.36 (9.71–11.07)		4.31 (3.25–5.79)	
		Only oral medication IR/non-medication IR		LAI IR/non-medication IR	
Overall	Reference	0.81 (0.80–0.83)	< 0.001	0.57 (0.52–0.63)	< 0.001
1 episode	Reference	0.77 (0.75–0.78)	< 0.001	0.57 (0.49–0.66)	< 0.001
2 episode	Reference	0.70 (0.67–0.73)	< 0.001	0.45 (0.37–0.55)	< 0.001
3 episode	Reference	0.66 (0.62–0.70)	< 0.001	0.37 (0.28–0.50)	< 0.001
4 or more episode	Reference	0.75 (0.70–0.80)	< 0.001	0.31 (0.23–0.42)	< 0.001
				LAI IR/only oral medication IR	
Overall		Reference		0.71 (0.64–0.78)	< 0.001
1 episode		Reference		0.74 (0.65–0.86)	< 0.001
2 episode		Reference		0.65 (0.53–0.79)	< 0.001
3 episode		Reference		0.56 (0.43–0.76)	< 0.001
4 or more episode		Reference		0.42 (0.31–0.56)	< 0.001

*LAI* long-acting injection, *SGA* second-generation antipsychotics, (95% confidence interval)

*IR* incidence rate $= \frac{\text{re - hospitalization events}}{{10 {\text{person - years}}}}$ (95% confidence interval)

$${\text{Only oral medication IR/non - medication IR}} = \frac{\text{Only oral medication incidence rate}}{\text{Non - medication incidence rate}}$$ (95% confidence interval)

$${\text{LAI IR}}/{\text{non - medication IR}} = \frac{\text{LAI incidence rate}}{\text{Non - medication incidence rate}}$$ (95% confidence interval)

$${\text{LAI IR/only oral medication IR}} = \frac{\text{LAI incidence rate}}{\text{Only oral medication incidence rate}}$$ (95% confidence interval)

**Fig. 2:**
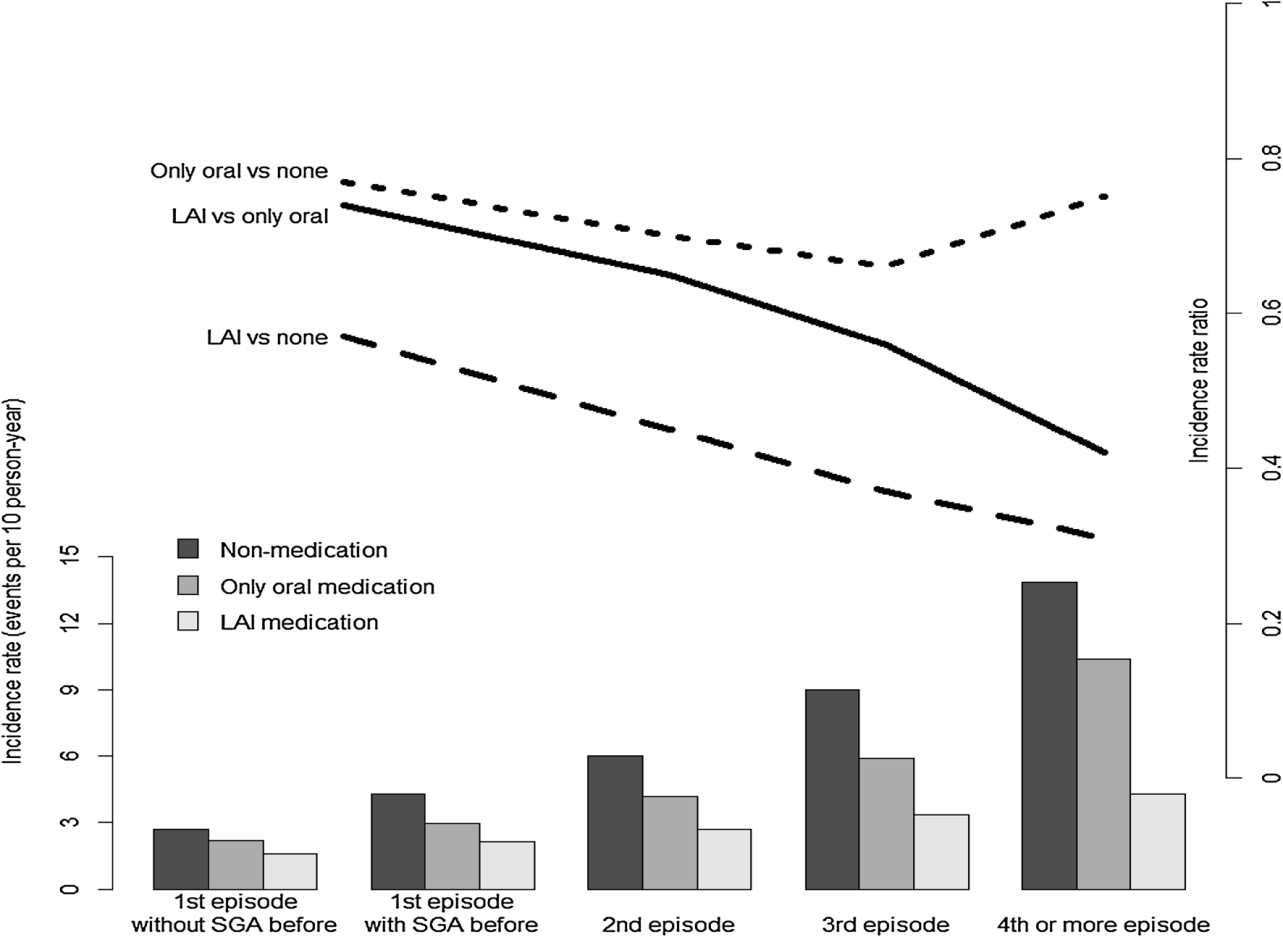
Incidence rate and incidence rate ratio during non-medication, oral medication alone, and long-acting injection (LAI) medication periods according to repeated admissions. *LAI* long-acting injection, *SGA* second-generation antipsychotics, ${\text{incidence rate}} = \frac{\text{re - hospitalization events}}{{10 {\text{person - years}}}}$, incidence rate ratio, ${\text{Only oral vs none}} = \frac{\text{Only oral medication incidence rate}}{\text{Non - medication incidence rate}}$, ${\text{LAI vs none}} = \frac{\text{LAI incidence rate}}{\text{Non - medication incidence rate}}$, ${\text{LAI vs only oral}} = \frac{\text{LAI incidence rate}}{\text{Only oral medication incidence rate}}$

The IRR for LAI versus oral medication alone at the first index admission was 0.74 (0.65–0.86). With repeated hospitalizations, this value changed to be more favorable to LAI. The IRR of the second, third, and fourth or more index admissions decreased by 0.65 (0.53–0.79), 0.56 (0.43–0.76), and 0.42 (0.31–0.56), respectively (Fig. 2).

## Discussion

Our study was a nationwide retrospective observational cohort study featuring within-subject comparisons. Claim databases are widely used in medical research including pharmacoepidemiological studies [9]. An advantage of claim databases is that they allow researchers to evaluate associations between specific drugs and events [10] that may be confounded by time-invariant variables, including genetic factors, chronic medical conditions, and patient lifestyle. However, it is difficult to adjust for time-invariant variables because medical databases often lack such information. In within-subject designs, each patient serves as their own control, thereby minimizing the confounding effects of time-invariant risk factors. Thus, the use of a within-subject analysis to investigate the real-world effectiveness of LAIs is the strength of our study.

LAIs offer an alternative to oral antipsychotics. Several second-generation LAI agents, including risperidone microspheres, paliperidone palmitate, aripiprazole monohydrate, and olanzapine pamoate monohydrate, are currently available. These agents were developed to improve treatment adherence and simplify the medication process [11]. Despite having several advantages, many clinicians have not routinely used LAIs due to fear of, or concern about, the injection, as well as concern over side effects, lack of insight, and high cost [12].

Although LAIs were developed to reduce the relapse rate in patients with schizophrenia, the findings of recent RCTs have called into question the benefit of LAIs over oral antipsychotics [7]. RCTs may not represent real-world settings, particularly in relation to schizophrenia. The patients who consent to participate in LAI clinical trials may not be representative of those who are prescribed LAIs in real-world settings. And clinical trial participants may be more adherent to a treatment, have less severe illness and better cognitive abilities to understand complex issues, and be more frequently monitored during clinical trials. All of which may attenuate the potential advantages of LAIs [7]. A meta-analysis of 21 RCTs found that LAIs and oral antipsychotics were similarly effective in preventing relapse at the longest time point (relative risk = 0.93, 95% CI 0.71–1.07, *P* = 0.31). Previous studies showing that fluphenazine-LAI was more effective than oral antipsychotics were all conducted prior to 1992 [7].

Our findings on the association between antipsychotics and readmission are consistent with those of a previous large observational study, which found that the risk of readmission was 20–30% lower in patients treated with LAIs compared with those receiving oral medication [9]. We found that LAIs lowered the readmission rate by 29% compared with oral antipsychotic medication, and by 43% in patients who had not received medication. Our large population sample contributed to the increased precision of our results, as indicated by the narrower CIs compared with a previous observational study [9].

Recurrence, characterized by acute psychotic exacerbation, is common in patients with schizophrenia. Repeated psychotic episodes may worsen psychopathology and social functioning [13]. The neurotoxicity hypothesis of psychosis suggests that active psychosis has a toxic effect on the brain and that acute psychotic exacerbations promote disease progression and impair the treatment response [14, 15]. A 7-year follow-up study found that 80% of patients with schizophrenia deteriorated over time and that the degree of deterioration was significantly correlated with the number of recurrence [16]. Our study design controlled for time-invariant confounders, but not for changes in the severity of the schizophrenia. Therefore, we analyzed the data according to each re-hospitalization to examine the effectiveness of LAIs on multiple episodes. With each hospitalization, the risk of readmission increased significantly under all of the medication conditions; however, the risk was highest during the no medication period.

Given that, the patients prescribed LAIs were more likely to have been non-adherent, and to have had a more severe disease status than those receiving oral medications, so LAIs significantly lowered the readmission rate compared with oral medication in real-world settings.

We found that the risk of readmission increased with repeated admissions, and that the benefit of LAIs was greater than that of oral antipsychotics in patients with multiple admissions. However, it is not clear whether the effect is due to heterogeneity of schizophrenia, reflected as a diverse spectrum of severity, or to blunting of the medication response by repeated exacerbations.

Our findings support those of a recent observational study suggesting that LAIs decrease the readmission rate [9]. Furthermore, we found that LAIs significantly reduced the risk of readmission, particularly in patients who experienced admissions in real-world settings. These findings warrant further research of each specific second-generation LAIs on the risk of readmission, to provide more detailed information of treatment options for targeting patients with multiple relapses.

Our study had several limitations. First, we assumed that refill compliance was an indicator of medication adherence, introducing a potential definition bias. Claims data provide information on drug prescriptions, but not patient adherence, which may have led to misclassification of the exposure periods. It is likely that some patients were not taking their prescribed medication during the periods we classified as medication-exposed, leading to a potential overestimation of adherence, particularly for oral medications. Therefore, our estimations of the medication and non-medication periods may have been inaccurate. Quantifying adherence is difficult because it is rarely an all or none phenomenon, and clinicians have a limited ability to identify patients who are not compliant [17]. However, despite limitations in evaluating medication adherence, drug prescriptions are widely used in large-scale population studies. Second, as with all claim-based studies, the data were collected for administrative purposes and may be subject to coding errors. Therefore, it is possible that the inclusion of false-positive patients who presented with schizophrenic symptoms led to an underestimation of the readmission rate during the non-medication period, although the most severe cases, i.e., patients who were readmitted 30 days after discharge, were excluded from the study. We included typical cases of schizophrenia characterized as a primary diagnosis of F20 Schizophrenia or F25 Schizoaffective disorder at index admission, undergoing treatment with SGAs and hospitalized between 7 and 120 days. Third, our analysis grouped the medications into oral and LAI agents rather than assessing the effectiveness of the individual drugs, which may have helped clinicians identify appropriate drugs for specific patients. Different drug agents have effects of varying magnitudes. Moreover, differences among second-generation LAIs have been observed in relation to the onset of clinical efficacy and the relationships between symptoms and functioning scores [18]. However, in general, the association between treatment and outcome is consistent among specific antipsychotics [19]. Finally, we enrolled patients with coexisting conditions and those who were taking concomitant medications. Nevertheless, these data are representative of a variety of real-world clinical settings in which patients with schizophrenia are treated.

LAIs lowered the readmission rate by 29% compared with oral antipsychotic medication in a real-world setting. The higher the readmission rate, the greater the benefit of LAIs in reducing the risk of re-hospitalization compared to oral antipsychotics. Our real-world findings strongly support the benefit of using LAIs over oral antipsychotics in schizophrenia patients with multiple recurrences by reducing 58% of readmissions.

Our result shows that clinicians may not hesitate to prescribe LAIs especially those with recurrent schizophrenia patients. Considering various expenses needed to those of individual patients and their families for hospital admissions, LAIs could relieve much financial burden. Lower readmission rate is one of the main health outcomes in mental health. This would allow decrease psychiatry inpatient beds and improve the quality of life for patients. There should be no limitation of usage or reimbursement of LAIs in case low compliance or high readmission probability is expected.

## Conclusions

This study found benefit of LAI treatment from preventing readmission of Schizophrenia patients. Although it is not clear in RCT studies, LAI may be more useful to patients with repeated admissions in real-world settings compared with only oral medication.

## Data Availability

Not applicable.

## Abbreviations

HIRA: Health Insurance Review and Assessment Service
IR: incidence rate
IRR: incidence rate ratio
LAI: long-acting injection
SGA: second-generation antipsychotics
